# Ocular Tics and Pediatric Autoimmune Neuropsychiatric Disorders Associated with Streptococcal Infections (PANDAS)

**DOI:** 10.3390/diseases12050083

**Published:** 2024-04-25

**Authors:** Stefano Dore, Daniele Satta, Angelo Zinellu, Giacomo Boscia, Arturo Carta, Mario Fruschelli, Rita Serra, Antonio Pinna

**Affiliations:** 1 Department of Medicine, Surgery, and Pharmacy, University of Sassari, 07100 Sassari, Italy; stefanodore@hotmail.com (S.D.); dan.satta@gmail.com (D.S.); rita.serra@ymail.com (R.S.); 2 Ophthalmology Unit, Azienda Ospedaliero Universitaria di Sassari, 07100 Sassari, Italy; 3 Department of Biomedical Sciences, University of Sassari, 07100 Sassari, Italy; azinellu@uniss.it; 4 Department of Ophthalmology, University of Turin, 10126 Turin, Italy; bosciagiacomo@gmail.com; 5 Ophthalmology Unit, Department of Medicine and Surgery, University of Parma, 43126 Parma, Italy; arturo.carta@unipr.it; 6 Ophthalmology Unit, Department of Medicine, Surgery and Neuroscience, University of Siena, 53100 Siena, Italy; mario.fruschelli@unisi.it

**Keywords:** oculomotor tics, PANDAS, ASO antibodies, anti-DNase B antibodies, ESR, CRP

## Abstract

Little is known about ocular tics in Pediatric Autoimmune Neuropsychiatric Disorders associated with Streptococcal infections (PANDAS). In this retrospective study, we examined the clinical records of children with motor tics referred to the Ophthalmology Unit, Azienda Ospedaliero-Universitaria di Sassari, Italy, in 2010–2019. The presence of ocular tics was investigated. Data about antistreptolysin O (ASO) and anti-DNase B antibody titers, erythrocyte sedimentation rate (ESR), plasma C-reactive protein (CRP), and antibiotic use were recorded. Forty children (thirty-four boys and six girls; mean age: 7.65 ± 2.5 years) with motor tics were identified; thirty-three (82.5%) showed ocular tics. Children with ocular tics had significantly higher titers of anti-DNase B antibodies (*p =* 0.04) and CRP (*p =* 0.016) than those with extraocular tics. A diagnosis of PANDAS was made in 24 (60%) children. PANDAS children with oculomotor tics had significantly higher titers of anti-DNase B antibodies (*p =* 0.05) than those with extraocular tics. Oral antibiotics were given to 25/33 (76%) children with ocular tics and 21/24 (87.5%) with PANDAS. All treated patients showed marked improvement/complete resolution of symptoms. Results suggest that higher titers of anti-DNase B antibodies may be implicated in the pathogenesis of ocular tics in PANDAS. Oral antibiotics may be beneficial in improving ocular tics. Further research is necessary to confirm our findings.

## 1. Introduction

Motor tics are rapid, involuntary, purposeless, repetitive, intermittent, and stereotyped muscle movements not associated with alterations in consciousness. The spectrum of ocular tics includes eyelid blinking, winking, eye rolling, and staring [1]. Eye tics occur intermittently and can, at least in part, be voluntarily suppressed.

PANDAS stands for Pediatric Autoimmune Neuropsychiatric Disorders associated with Streptococcal infections. This uncommon condition is clinically characterized by obsessive–compulsive disorders (OCDs) and/or tics, particularly multiple, complex, or unusual tics. It usually occurs in children between 3 and 12 years of age with infections caused by group A β-hemolytic streptococci, such as sinusitis, tonsillitis, and scarlet fever. PANDAS may have an acute onset or show an episodic, relapsing–remitting course. It is often associated with neuropsychiatric symptoms, including emotional lability, anxiety and/or depression, irritability, aggression, severe oppositional behaviors, developmental regression, abrupt deterioration in school performance (e.g., dramatic handwriting changes, decline in orthographic memory, difficulties in mathematics), and sleep disturbances [2,3,4]. Enuresis or urinary frequency may also occur, often erroneously diagnosed as a urinary tract infection when other typical neurological symptoms are not present. Children with PANDAS are described as “completely new and unrecognizable” by their shocked parents and/or caregivers [5].

Clinical and laboratory evidence suggests that PANDAS is a streptococcal-induced autoimmune disease. This condition is believed to be etiologically similar to Sydenham’s Chorea, a manifestation of acute rheumatic fever (ARF) [2]. About 30% of ARF patients have Sydenham’s chorea; of these, approximately 70% will develop OCDs. Sydenham’s chorea and PANDAS are thought to share a common pathophysiological mechanism, involving the molecular mimicry of group A β-hemolytic streptococcal bacteria, which stimulates the production of antibodies cross-reacting with antigens in the brain [3]. This autoimmune response may induce a variety of neurologic and psychiatric signs and symptoms, typically observed in patients with Sydenham’s chorea and PANDAS [3].

Antideoxyribonuclease-B (anti-DNase B) and antistreptolysin O (ASO) are the main antibodies produced in response to group A β-hemolytic *Streptococcocus* infections. Their measurement is used for the laboratory diagnosis of acute or recent-onset infection caused by this organism. Anti-DNase B antibodies typically remain elevated longer than ASO and may remain higher for several months after infection.

A PANDAS diagnosis is clinical, requiring a detailed medical history and a full physical examination, while management is complex. Untreated or unrecognized PANDAS may increase the risk of OCDs during adulthood.

To the best of our knowledge, an association between streptococcal infection and ocular tics has only been documented in PANDAS [2]. Little is known about ocular tics in PANDAS. The purpose of this retrospective study was to investigate ocular tics in a cohort of children with motor tics and explore their correlation with PANDAS, anti-DNase B and ASO antibody titers, and some known markers of inflammation, such as erythrocyte sedimentation rate (ESR) and plasma C-reactive protein (CRP).

## 2. Materials and Methods

In this retrospective study, the clinical records of all children with motor tics referred by pediatric neurologists and neuropsychiatrists to the Ophthalmology Unit, Azienda Ospedaliero-Universitaria di Sassari, Sassari, Italy, between 2010 and 2019 were examined. Both children with ocular and non-ocular tics were involved.

Ethical review and approval were waived for this study due to this was a retrospective study performed on already available data. The study was conducted in full accord with the tenets of the Declaration of Helsinki. The children’s parents received detailed information and provided informed consent via telephone before inclusion.

The detection of ocular tics was investigated. For this survey, ocular tics were defined as (1) eyelid blinking, eye rolling, and eye widening; (2) purposeless, repetitive, and brief (lasting few seconds) movements of the eyelids or eyeballs not associated with alterations in consciousness; and (3) involuntary in nature, but sometimes partially suppressible. Children with excessive blinking secondary to ocular allergy or conjunctivitis were excluded.

Data about plasma ASO and anti-DNase B antibody titers, ESR, CRP, and antibiotic use were recorded and statistically analyzed.

A diagnosis of PANDAS was made if the following criteria were fulfilled: (1) presence of OCDs, attention deficit hyperactivity disorder (ADHD)-like symptoms, and/or tics, especially multiple, complex, or unusual tics; (2) symptom onset between three years of age and puberty; (3) acute onset and episodic, relapsing–remitting course; (4) association with group A β-hemolytic streptococcal infection, confirmed by culture or the detection of ASO and anti-DNase B antibodies; (5) association with neurological abnormalities, including anxiety, emotional lability, motor hyperactivity, bedwetting, choreiform movements, mood changes, or developmental regression (PANDAS diagnostic flow chart; PANDAS Physician Network ©2020, available at: www.pandasppn.org/flowchart (accessed on 31 March 2024)). By definition, PANDAS is a diagnosis of exclusion, after elimination of all other possible causes.

Statistical analysis was performed using Fischer’s exact test, Student’s *t* test, or the Krustal–Wallis test, when appropriate. Spearman’s rank correlation test was used to test for correlations between ASO and anti-DNase B antibody titers, ESR, and CRP. *p* values ≤ 0.05 were considered to be statistically significant. Statistical analysis was performed with commercial software (STATA ver. 9.0; StataCorp, College Station, TX, USA).

## 3. Results

### 3.1. Children with Motor Tics

Forty children (thirty-four boys, six girls; mean age at onset: 7.65 ± 2.5 years) with motor tics were identified. Thirty-three (82.5%) showed ocular tics, including eyelid blinking (25), eye rolling (3), eye widening (3), gaze deviation (4), and saccadic intrusions (1); five of them had multiple ocular tics.

Demographics, a family history of tics, psychiatric and autoimmune disorders, simple motor tics, complex tics, facial tics, comorbidities, and diagnosis of PANDAS are presented in Table 1.

Most children (92.5%) had a negative family history of tics. A positive family history of psychiatric disorders, including anxiety, depression, bipolar syndrome, schizophrenia, specific learning disabilities (SLDs), and ADHD, was present in 42.5% of children. A positive family history of autoimmune disorders, including type 1 diabetes mellitus, Hashimoto’s thyroiditis, rheumatoid arthritis, myasthenia gravis, multiple sclerosis, ankylosing spondylitis, and thrombocytopenic purpura, was observed in 40% of children. Simple motor tics (i.e., movements involving only one muscle group or body part) were present in 95% of patients. Complex motor tics (i.e., movements involving multiple muscle groups and appearing as semi-purposeful movements) were observed in 70% of them. Twenty-three (57.5%) patients had facial tics, including grimacing, nose scrunching, lip and tongue biting, lip twitching, and tongue protrusion. Eighteen (45%) children had comorbidities, including SLDs, hepatobiliary disorders, epilepsy, Wilson’s disease, partial deafness, hyperfibrinogenemia, Down’s syndrome, congenital ichthyosis, and benign tibial bone lesion.

Anti-DNase B antibody titers were available for 35 patients. The median value was 0 (range 0–580) IU/mL in children with oculomotor tics and 0 (range 0–0) IU/mL in children with extraocular tics, a statistically significant difference (*p* = 0.04).

CRP values were available for 37 patients. The median CRP was 0.35 (range 0.26–0.4) mg/dL in children with oculomotor tics and 0.14 (range 0.11–0.25) mg/dL in children with extraocular tics, again a statistically significant difference (*p* = 0.04).

Conversely, the median ASO antibody titer was 406 (range 171–564) IU/mL in children with oculomotor tics and 343 (range 53–482) IU/mL in children with extraocular tics, which was not a statistically significant difference (*p* = 0.51).

ESR values were available for 38 patients. The median ESR was 9 (range 4.5–20.25) mm/h in children with oculomotor tics and 4 (range 2.25–7.5) mm/h in children with extraocular tics, a difference which fell just short of statistical significance (*p* = 0.06).

**Figure 1:**
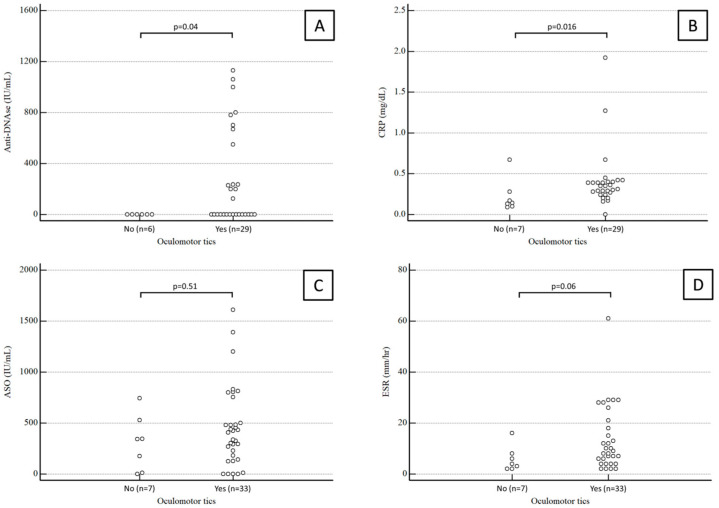
Anti-DNase B antibody titers (**A**), CRP (**B**), ASO antibody titers (**C**), and ESR (**D**) in children with and without ocular tics.

Results about anti-DNase B antibody titers, CRP, ASO antibody titers, and ESR in children with and without ocular tics are summarized in Figure 1.

Not surprisingly, Spearman’s rank correlation test showed a statistically significant positive correlation between anti-DNase B and ASO antibody titers (rho = 0.448; *p* = 0.0069).

Oral antibiotics (macrolides or β-lactams) had been given for 2–6 weeks to 25 (76%) out of 33 children with ocular tics. All the treated patients showed marked improvement or complete resolution of symptoms; conversely, no improvement was observed in those untreated. In eight (32%) children, there was recurrence of ocular tics after completion of antibiotic therapy.

### 3.2. PANDAS Children

A diagnosis of PANDAS was made in a subset of 24 (60%) children (19 boys, 5 girls; mean age: at onset 7.25 ± 2.2 years). Twenty (83.3%) had ocular motor tics, including eyelid blinking (16), eye rolling (2), eye widening (2), and gaze deviation (2). In two cases, there were multiple ocular tics (e.g., eyelid blinking and gaze deviation).

All PANDAS children had positive ASO antibodies, whereas the presence of anti-DNase B antibodies was observed in 19 out of 24. Ten had a positive throat culture for group A β-hemolytic streptococcus. Demographics, a family history of tics, psychiatric and autoimmune disorders, simple motor tics, complex tics, facial tics, and comorbidities in children with PANDAS are shown in Table 2.

Most children (87.5%) had a negative family history of tics. A positive family history of psychiatric disorders, including anxiety, depression, bipolar syndrome, and SLDs, was present in 33% of children. A positive family history of autoimmune disorders, including type 1 diabetes mellitus, Hashimoto’s thyroiditis, rheumatoid arthritis, myasthenia gravis, multiple sclerosis, ankylosing spondylitis, and thrombocytopenic purpura was observed in 50% of children. Seven (29%) of them had comorbidities, including hepatobiliary disorders, epilepsy, Wilson’s disease, partial deafness, and SLDs.

Anti-DNase B antibody titers were available for 19 patients. The median value was 234 (range 0–750) IU/mL in PANDAS children with oculomotor tics and 0 (range 0–0) IU/mL in PANDAS children with extraocular tics, a statistically significant difference (*p* = 0.05). 

Conversely, the median ASO antibody titer was 482 (range 320–809) IU/mL in PANDAS children with oculomotor tics and 351 (range 93–636) IU/mL in PANDAS children with extraocular tics, not a statistically significant difference (*p* = 0.49).

CRP values were available for 21 patients. The median CRP was 0.35 (range 0.26–0.4) mg/dL in PANDAS children with oculomotor tics and 0.14 (range 0.11–0.25) mg/dL in PANDAS children with extraocular tics, again not a significant difference (*p* = 0.49).

ESR values were available for 23 patients. The median ESR was 4.5 (range 8–12.75) mm/h in PANDAS children with oculomotor tics and 7 (range 4–12) mm/h in PANDAS children with extraocular tics, not a statistically significant difference (*p* = 0.6). 

**Figure 2:**
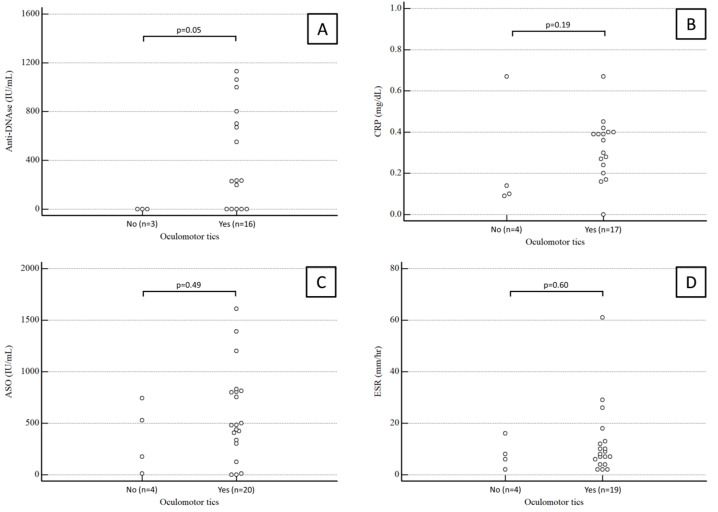
Anti-DNase B antibody titers (**A**), CRP (**B**), ASO antibody titers (**C**), and ESR (**D**) in PANDAS children with and without ocular tics.

Results for anti-DNase B antibody titers, CRP, ASO antibody titers, and ESR in PANDAS children with and without ocular tics are summarized in Figure 2.

Spearman’s rank correlation test failed to find any statistically significant correlation between ASO and anti-DNase B antibody titers, ESR, and CRP.

Twenty-one (87.5%) out of twenty-four children with PANDAS had received oral antibiotics for 2–6 weeks. All of them showed marked improvement or complete resolution of ocular tics. However, in seven (29%), there was a recurrence of symptoms after antibiotic therapy. The clinical characteristics of these patients are described in Table 3.

## 4. Discussion

Motor tics are probably the most prevalent movement disorder in childhood. The reported prevalence of tics varies widely. In a recent meta-analysis, tic disorders showed a prevalence rate of approximately 3% in children between the ages of 6 and 15 years [6]. Their onset usually occurs between two and seven years of age, with the worst period of expression peaking during pre-puberty (9 to 12 years), followed by a phase of stabilization and attenuation in adolescence or early adulthood. In most cases, the first tics are oculomotor. Approximately 40% of children will have no tics during adulthood, 40% will show minimal or mild tics causing no interference in their lives, and 20% will continue to have moderate/severe symptoms [5].

Frequent eye blinking and rolling are a common cause for referral to pediatric ophthalmologists and neurologists. Most articles on ocular tics in children have focused on the characterization and management of this disorder in Tourette’s syndrome, a relatively frequent neuro-developmental disorder beginning in childhood or teenage years and presenting with multiple oculomotor tics and at least one vocal tic, OCDs, and ADHD [7]. Martino et al. have found that nearly 95% of 212 adult and pediatric patients with Tourette’s syndrome had ocular tics [8]. On the other hand, little is known about ocular tics in PANDAS.

The first description of PANDAS dates to 1998, when Swedo et al. [2] published the landmark paper on this topic and provided guidelines for diagnosis. In a more recent article published in 2004 [9], these were updated and clarified. The Guidelines and Therapeutics Committee of the PANDAS Physician Network (PPN) has now provided a guideline for making the diagnosis of PANDAS based upon the above-mentioned articles (PANDAS diagnostic flow chart; PPN ©2020, available at: www.pandasppn.org/flowchart (accessed on 31 March 2024)) [2,9]. 

The symptoms of PANDAS first become evident between 3 years of age and puberty. Early onset is related to times of peak exposure (early grade school years) and the development of cross-species immunity in response to group A β-hemolytic streptococcal infections due to the production of antibodies against the conserved portion of the streptococcal M-protein [10]. These antibodies are demonstrated in 98% of 12-year-old youth with former group A β-hemolytic streptococcal infection [10,11]. However, the other 2% would remain vulnerable to post-group A β-hemolytic streptococcal sequelae, including PANDAS and rheumatic fever. Post-pubertal onset of PANDAS is rare but also possible [9].

The clinical course of PANDAS may have an acute onset or be relapsing–remitting. In the acute pattern, there is an abrupt, dramatic onset of OCD and/or tics. Comorbid neuropsychiatric symptoms are always present. In the relapsing–remitting pattern, there are dramatic, debilitating exacerbations of existing symptoms. While tics are known to worsen when the child suffers from an infection, for this criterion to be met, tic exacerbation must incapacitate the patient—often suddenly precluding them from attending school or requiring an examination at the emergency department. In both patterns, the severity of symptoms usually decreases significantly between episodes, but occasionally does not remit to pre-onset levels. Furthermore, since children are consistently exposed to pathogens at school or with siblings, and since post-onset immune responses to viral and bacterial infections (not just group A β-hemolytic streptococci) can trigger exacerbations, the symptomatic periods may be protracted, but may vary from case to case.

Symptom exacerbations must be temporally related to group A β-hemolytic streptococcal infections (i.e., associated with a positive throat culture and/or elevated ASO or anti-DNase B antibodies). In PANDAS, group A β-hemolytic streptococcal infections often are found without apparent sore throat. Rising titers of anti-DNase B and ASO antibodies can be used to indicate a former streptococcal infection. However, there are several issues associated with using titers: (1) titers from a prior streptococcal infection may remain high for many months in some children, creating a potential false-positive association; (2) 40% of children with documented group A β-hemolytic streptococcal infections do not have a titer increase, creating a potential false negative; (3) timing is critical in looking for the 2- to 4-fold rise in titer (1 to 4 weeks for ASO from initial infection and 6 to 8 weeks for anti-DNase B). If a child is seen within a few days of symptom onset and has a negative throat culture, it may be useful to check baseline anti-streptococcal antibody titers and then obtain a second set of titers 6 to 8 weeks later and look for a 2- to 4-fold increase, which is suggestive or indicative of a recent infection.

The diagnostic criteria for PANDAS only require the presence of OCDs or tics, but comorbid neuropsychiatric symptoms are universally present [2,9]. During symptom exacerbations, PANDAS children will have abnormal results on neurological examination. Motor hyperactivity and adventitious movements, including tics or choreiform movements are very common. It is of critical importance to distinguish between PANDAS and Sydenham’s chorea, since the latter is a known manifestation of rheumatic fever and has its own distinct characteristics and treatment regimen.

Although the exact pathophysiological mechanism underlying PANDAS is still unclear [12], an association with a former o concomitant group A β-hemolytic streptococcal infection has been well documented [2]. Clinical and experimental evidence indicates that PANDAS is an autoimmune disorder. Streptococcal infections may cause the production of cross-reactive antibodies which penetrate the blood–brain barrier, bind to neuronal antigens, and dysregulate basal ganglia functions, thus determining the onset of the neuropsychiatric symptoms [13]. Neurons connecting to the basal ganglia affect motor function, emotion, behaviors, procedural learning, cognition, and sensory issues. In the brain, anti-neuronal streptococcal-induced autoantibodies bind to specific antigens, including dopamine receptors [14], lysoganglioside [15,16], β-tubulin [17], and calcium-calmodulin dependent protein kinase II (CaMKII) [16]. Furthermore, neuroglial immune cells in the brain may incite basal ganglia inflammation [18].

Experimental mouse models have shown that human anti-neuronal streptococcal-induced autoantibodies targeting dopamine D2 receptor (D2R) and CaMKII cause obsessive behaviors like those seen in children with PANDAS [14,19,20]. Accordingly, several studies have tried to identify potential biomarkers which may be useful to confirm the diagnosis of PANDAS [21,22,23]. Unfortunately, the results of these investigations have been inconclusive and failed to find a significant correlation between symptom recurrence and the detection of specific autoantibodies. Therefore, in a recent review, Dale has suggested that PANDAS may be the result of a heterogeneous dysregulation of the immune system involving immune-associated genes, immunoglobulins, T-lymphocytes, cytokines, and cerebrospinal fluid oligoclonal bands [24]. In this regard, Chain et al. [25] have recently reported elevated titers of IgG specific for neuronal autoantigens and increased CaMKII activity in human neuronal cells during acute PANDAS, whereas a decrease in autoantibody titers during improvement has been documented.

So far, there has been no established biomarker for PANDAS; however, ESR, CRP, and the detection of plasma ASO and anti-DNase B antibodies have been recommended in suspected cases [21,26]. Nevertheless, it is important to keep in mind that the finding of positive anti-streptococcal titers only indicates exposure to the streptococcal antigens and do not distinguish between the state of carrier and acute infection [3,26].

In our retrospective study on a cohort of 40 children with motor tics, 33 (82.5%) showed ocular tics. Children with oculomotor tics had significantly higher plasma values of anti-DNase B antibodies (*p* = 0.04) and CRP (*p* = 0.016) than those with extraocular tics. 

Little is known about the prevalence of PANDAS among children with tics. In our study, a diagnosis of PANDAS was made in 24 (60%) out 40 children with motor tics referred by pediatric neurologists and neuropsychiatrists to our Unit. Conversely, in the report by Catarina Prior et al. [27] only five (6.4%) out seventy-eight children with motor tics had PANDAS. This discrepancy may be explained by differences in data collection from clinical records.

Boys with PANDAS outnumbered girls by a ratio of 3.8:1, a result consistent with those reported by Swedo et al. (2.6:1) [2], Lepri et al. (2.9:1) [4], and La Bella et al. (2.8) [28]. Mean age at onset was 7.25 years, a value similar to those found by the same authors (6.3, 6.4, and 7 years, respectively) [2,5,28] and by Rea et al. (6.2 years) [29].

A positive family history of psychiatric disorders, including anxiety, depression, bipolar syndrome, and SLDs, was present in 33% of PANDAS children. Likewise, Rea et al. [29] reported the presence of neuropsychiatric disorders in 40% of the relatives (parents, siblings, grandparents, and uncles) of children with PANDAS.

A positive family history of autoimmune disease was found in 42.5% of children with motor tics and in 50% of PANDAS children, a finding consistent with the study by O’Dor et al. [30], who reported approximately 30% of mothers endorsing one or more autoimmune conditions and hypothesized a correlation between maternal autoimmune disease and PANDAS in youth.

Twenty (83.3%) children had ocular tics, including eyelid blinking, eye rolling, eye widening, and gaze deviation, a result consistent with the report by La Bella et al. [28], who found tics involving the eyes in eleven (61%) out of eighteen children with PANDAS. In this subset of patients, we documented significantly higher plasma values of anti-DNase B antibodies (*p* = 0.05). Thus, there arises the interesting question of whether, or not, higher titers of this antistreptococcal antibody may primarily cause involuntary eyelid and eye movements.

Treating PANDAS may include antibiotics, steroids, nonsteroidal anti-inflammatory drugs (NSAIDs), intravenous administration of immunoglobulins (IVIG), plasmapheresis cognitive–behavioral therapy (CBT), and selective serotonin reuptake inhibitors (SSRIs) [31,32,33,34,35,36,37]. 

Emerging clinical data have shown therapeutic benefits of antibiotics (macrolides or β-lactams) in children with PANDAS [3,5,33,34,35]. An initial treatment for three weeks has been recommended, awaiting resolution of neuropsychiatric symptoms. Response to antibiotics can occur quickly, with complete or partial remission of OCDs, anxiety, and many of the comorbid symptoms of PANDAS within 24–48 h. More often, however, the response occurs later, usually after one or two weeks of therapy. If no improvement is seen after a fortnight, an alternate class of antibiotics for an additional treatment of 10–14 days should be considered. If the antibiotics produce significant improvement in symptoms, they may be continued at treatment level doses for an additional 2–4 weeks.

PANDAS children with a documented streptococcal throat infection should undergo a follow-up swab a week after completing antibiotic treatment. Re-treatment is recommended if the culture is still positive. Following the initial treatment course, prophylactic antibiotics may be useful for PANDAS. If the decision is made to use prophylactic antibiotics, the dosage and choice of antibiotics can be guided by the recommendations for prophylaxis in acute rheumatic fever. If there is symptom recurrence at a lower prophylactic dose, the dose may need to be adjusted. If a child shows a poor response, or continues to have frequent exacerbations, his/her family members should be examined and tested for group A β-hemolytic streptococcal infections. Recurrent exposure to group A β-hemolytic streptococci can trigger symptoms in PANDAS children, even if they do not develop a full-blown infection.

In our cohort, oral antibiotics were given for 2–6 weeks to 25 (76%) out of 33 children with ocular tics and 21 (87.5%) out of 24 children with PANDAS. In both groups, there was a marked improvement or complete resolution of ocular tics in all the children treated. After completion of antibiotic therapy, recurrence of symptoms was observed in eight (32%) children with ocular tics and in seven (29%) patients with PANDAS. The latter group was notable for having a positive family history of autoimmune disease, ocular tics, and complex motor tics at onset; however, the numbers are too small to hypothesize any kind of predisposing factor for symptom recurrence after antibiotic therapy.

In a recent Italian study involving 345 children with PANDAS [5], 75% showed an improvement in neurologic symptoms after prophylaxis with benzathine benzylpenicillin. This favorable outcome was reported to occur 3–5 months after starting antibiotics. 

It is unclear whether the improvements observed after antibiotic therapy may depend on treatment of a latent infection or some other non-antimicrobial-related effect [38]. According to Lepri et al. [5], long-term antibiotic prophylaxis may be considered the gold-standard for PANDAS management. 

Our study has several limitations, including the relatively small sample size, its retrospective nature, and the lack of a healthy control group. Data were collected from the patients’ records and laboratory investigations available, which may have resulted in a lack of systematic collection with some data missing, as highlighted in the Figures. The most important limitation is that this was not a randomized prospective investigation in which the patients were randomly distributed between a study group and a control group. We considered conducting a multivariate correlation to correct for various confounding factors. However, owing to the limited sample size and some missing data among the variables studied further reducing the number of observations, we decided not to use multivariate models to prevent the problem of overfitting, as recommended by the guidelines [39]. Despite these limitations, our goal was to describe our experience on PANDAS-related ocular tics, a relatively uncommon condition. 

A quarter of a century has passed since PANDAS was first described [2]. However, there are still unmet needs concerning a better definition of its clinical manifestations, the identification of specific biomarkers, and the more appropriate management. Although many children with PANDAS will have an excellent response to antibiotic treatment, some may develop persistent neuropsychiatric symptoms or have worsening symptoms after each recurrence of group A β-hemolytic streptococcal infection. Unrecognized or untreated manifestations of PANDAS can increase the risk of obsessive–compulsive manifestations and tics during adulthood.

In conclusion, ophthalmologists should be aware that the onset of ocular tics not related to ocular surface disorders in children may be the first manifestation of PANDAS. If PANDAS is suspected, children should be promptly referred to a pediatric neurologist and neuropsychiatrists. Our results suggest that higher plasma values of anti-DNase B antibodies may play a role in the pathophysiological mechanisms underlying ocular tics in PANDAS. Oral antibiotics (macrolides or β-lactams) may be beneficial in improving ocular tics, but recurrence of symptoms after completion of antibiotic therapy may occur. Further research is necessary to confirm our findings.

## Figures and Tables

**Table 1:** Age, gender, family history of tics, psychiatric and autoimmune disorders, simple motor tics, complex tics, facial tics, comorbidities, and diagnosis of PANDAS in a cohort of 40 children with motor tics (^§^ Student’s *t* test; * Fischer’s exact test).

	All Patients (n = 40)	Children with Ocular Tics (n = 33)	Children without Ocular Tics (n = 7)	*p*-Value
Age (years) ± SD	7.65 ± 2.45	7.63 ± 2.55	7.71 ± 2.14	0.94 ^§^
Gender (males/females)	34/6	28/5	6/1	1.00 *
Family history of tics (no/yes)	37/3	30/3	7/0	1.00 *
Family history of psychiatric disorders (no/yes)	23/17	20/13	3/4	0.43 *
Family history of autoimmune disorders (no/yes)	24/16	18/15	6/1	0.21 *
Simple motor tics (no/yes)	2/38	0/33	2/5	0.03 *
Complex motor tics (no/yes)	12/28	11/22	1/6	0.65 *
Facial tics (no/yes)	17/23	14/19	3/4	1.00 *
Comorbidities (no/yes)	22/18	18/15	4/3	1.00 *
PANDAS diagnosis (no/yes)	16/24	13/20	3/4	1.00 *

**Table 2:** Age, gender, family history of tics, psychiatric, and autoimmune disorders, simple motor tics, complex tics, facial tics, and comorbidities in a cohort of 24 PANDAS children with motor tics (^§^ Student’s *t* test; * Fischer’s exact test).

	All Patients (n = 24)	Children with Ocular Tics (n = 20)	Children without Ocular Tics (n = 4)	*p*-Value
Age (years) ± SD	7.25 ± 2.2	7.35 ± 2.18	6.75 ± 2.36	0.63 ^§^
Gender (males/females)	19/5	16/4	3/1	1.00 *
Family history of tics (no/yes)	21/3	17/3	4/0	1.00 *
Family history of psychiatric disorders (no/yes)	16/8	14/6	2/2	0.58 *
Family history of autoimmune disorders (no/yes)	12/12	8/12	4/0	0.93 *
Simple motor tics (no/yes)	2/22	0/20	2/2	0.02 *
Complex motor tics (no/yes)	5/19	5/15	0/4	0.54 *
Facial tics (no/yes)	12/12	9/11	3/1	0.59 *
Comorbidities (no/yes)	17/7	13/7	4/0	0.28 *

**Table 3:** Age, gender, family history of tics, psychiatric, and autoimmune disorders, simple motor tics, complex tics, facial tics, oculomotor tics, and comorbidities in PANDAS children who had symptom recurrence after antibiotic therapy.

	Patients (n = 7)
Age (years) ± standard deviation	7 ± 1.9
Gender (males/females)	6/1
Family history of tics (no/yes)	7/0
Family history of psychiatric disorders (no/yes)	7/0
Family history of autoimmune disorders (no/yes)	0/7
Simple motor tics (no/yes)	0/7
Complex motor tics (no/yes)	0/7
Facial tics (no/yes)	2/5
Oculomotor tics (no/yes)	0/7
Comorbidities (no/yes)	3/4

## Data Availability

Data are available on request.
